# Supplementary material for: Sn(IV)-free tin perovskite films realized by in situ Sn(0) nanoparticle treatment of the precursor solution
Source: Nat Commun. 2020 Jun 16;11:3008. doi: 10.1038/s41467-020-16726-3 (PMC7297727; doi:10.1038/s41467-020-16726-3)
Supplement: Supplementary file 1 — Supplementary Information [file 41467_2020_16726_MOESM1_ESM.pdf]

Supplementary Information

Sn(IV)-free tin perovskite films realized by *in situ* Sn(0) nanoparticle treatment of the precursor solution

Tomoya Nakamura,[1] Shinya Yakumaru,[1] Minh Anh Truong,[1] Kyusun Kim,[1] Jiewei Liu,[1] Shuaifeng Hu,[1] Kento Otsuka,[1] Ruito Hashimoto,[1] Richard Murdey,[1] Takahiro Sasamori,[2] Hyung Do Kim,[3] Hideo Ohkita,[3] Taketo Handa,[1] Yoshihiko Kanemitsu[1] & Atsushi Wakamiya[1*]

[1]Institute for Chemical Research, Kyoto University, Gokasho, Uji, Kyoto 611-0011, Japan.

[2]Graduate School of Natural Sciences, Nagoya City University, Yamanohata 1, Mizuho-cho, Mizuho-ku, Nagoya, Aichi 467-8501, Japan.

[3]Department of Polymer Chemistry, Graduate School of Engineering, Kyoto University, Katsura, Nishikyo-ku, Kyoto 615-8510, Japan.

*email: wakamiya@scl.kyoto-u.ac.jp

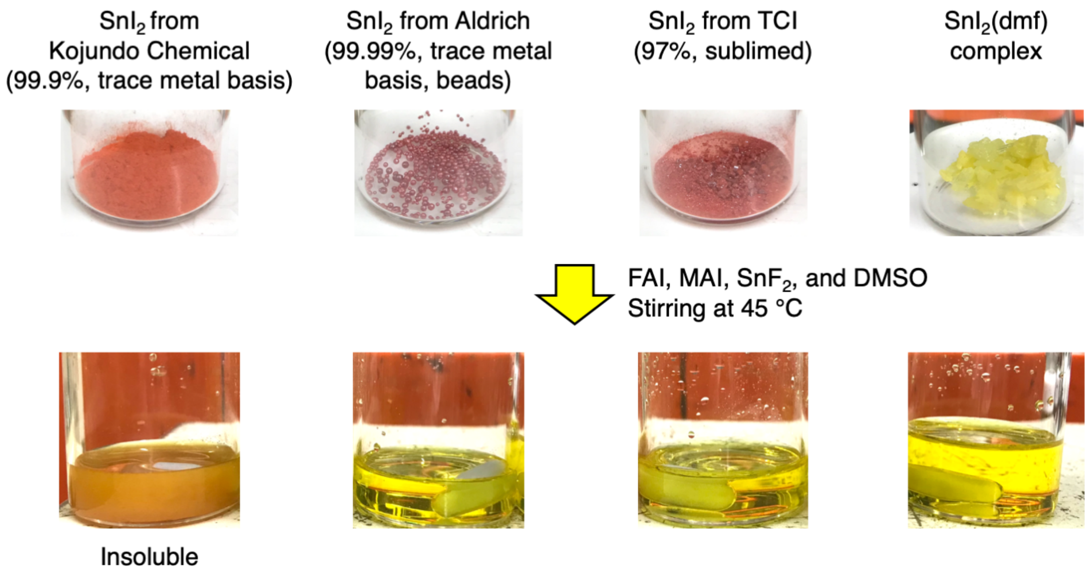

Supplementary Figure 1. Photos of SnI_2 sources and 0.9 M $FA_{0.75}MA_{0.25}SnI_3$ solutions with 10 mol% SnF_2 in DMSO using each SnI_2 source.

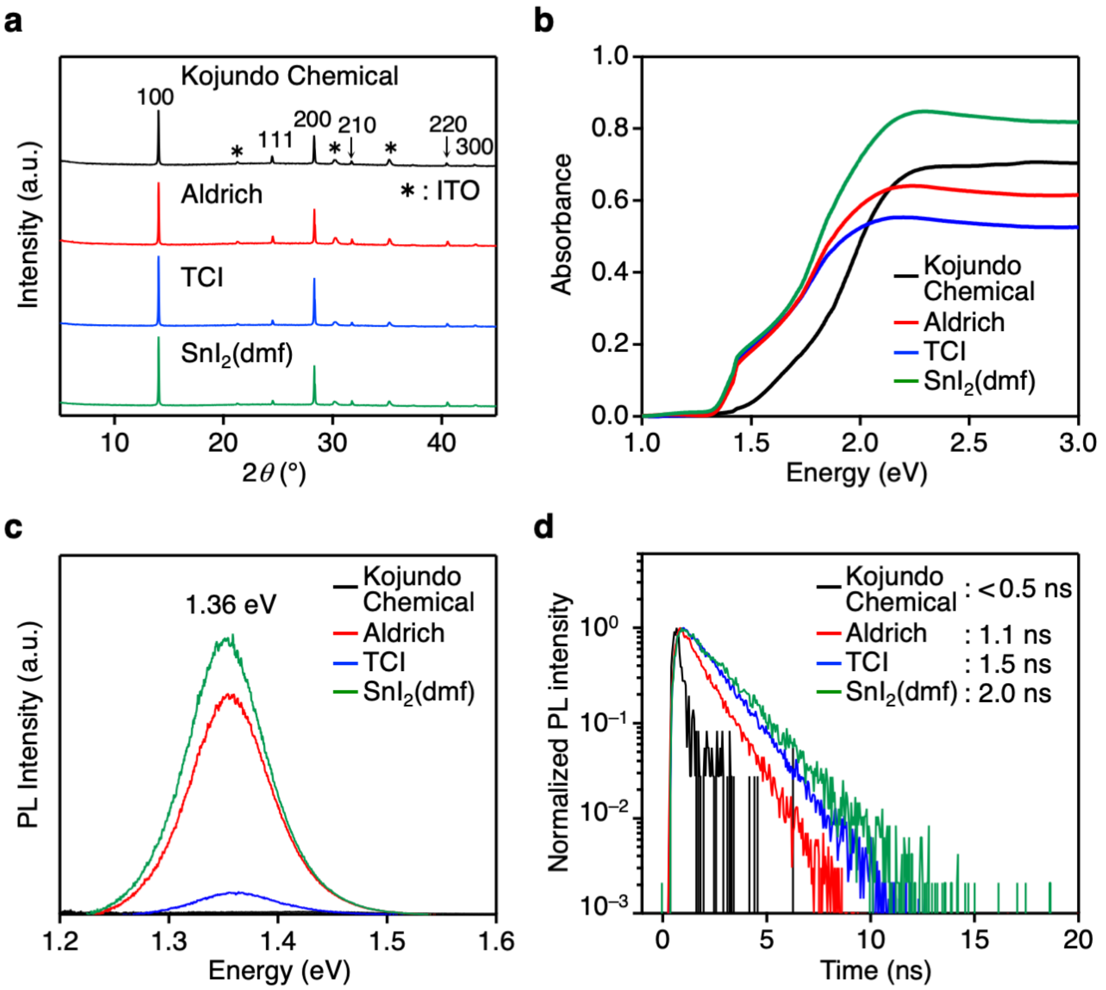

Supplementary Figure 2. Characteristics of the Sn-perovskite prepared from different SnI_2 sources. **a** XRD, **b** UV–vis–NIR absorption, **c** PL, and **d** TRPL spectra.

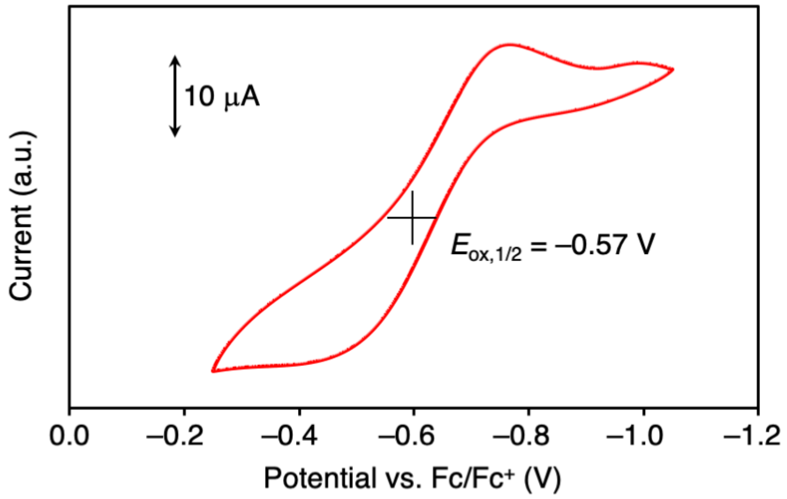

Supplementary Figure 3. Cyclic voltammogram of TM-DHP, recorded in CH_2Cl_2 (1 mM) at a scan rate of 100 mV s^{-1} using n-Bu_4NPF_6 (0.1 M) as the supporting electrolyte.

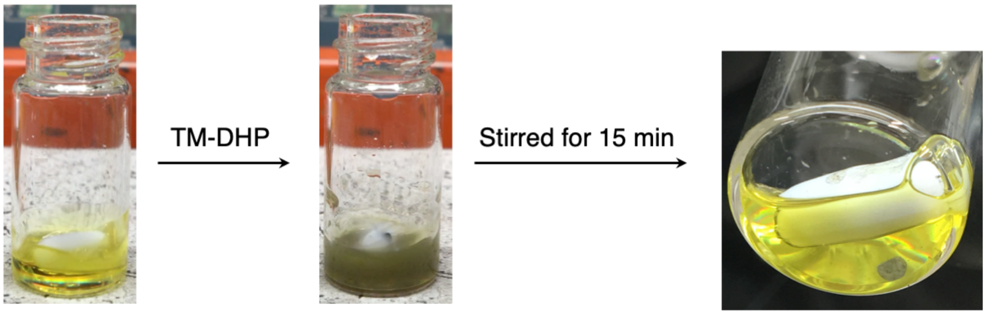

Supplementary Figure 4. Photos of the perovskite precursor solution before (left), just after the addition of TM-DHP (middle), and after stirring for 15 min (right).

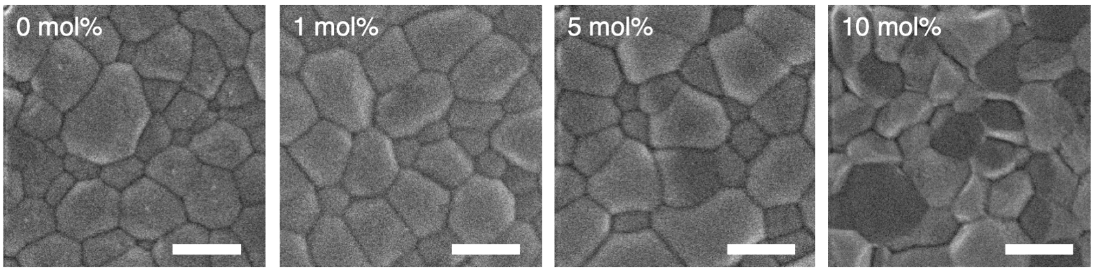

Supplementary Figure 5. Top-view scanning electron microscopy (SEM) images of $FA_{0.75}MA_{0.25}SnI_3$ perovskite films prepared with 0, 1, 5, and 10 mol% TM-DHP together with 10 mol% SnF_2. The scale bar is 500 nm.

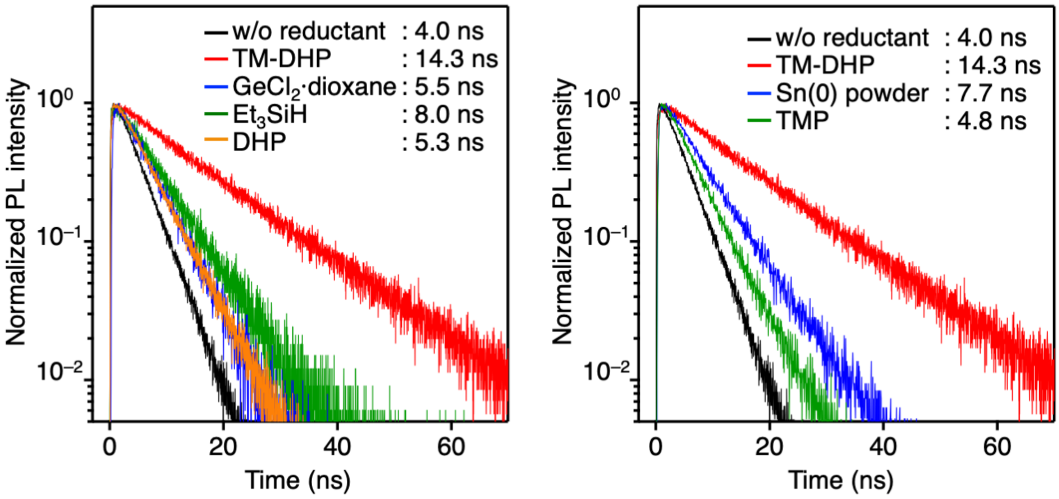

Supplementary Figure 6. PL decay curves of $FA_{0.75}MA_{0.25}SnI_3$ perovskite films prepared with or without 1 mol% of various additives together with 10 mol% SnF_2.

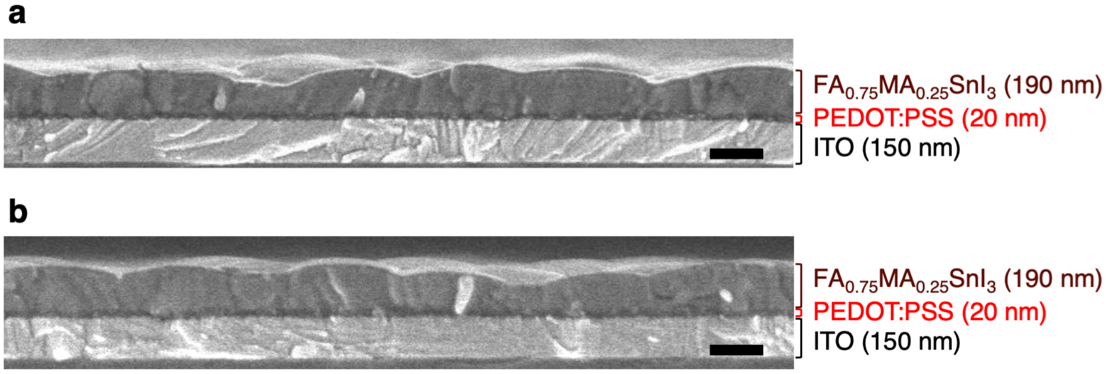

Supplementary Figure 7. Cross-sectional SEM images of $FA_{0.75}MA_{0.25}SnI_3$ perovskite films. The films were prepared **a** with or **b** without 1 mol% of TM-DHP. The scale bar is 200 nm.

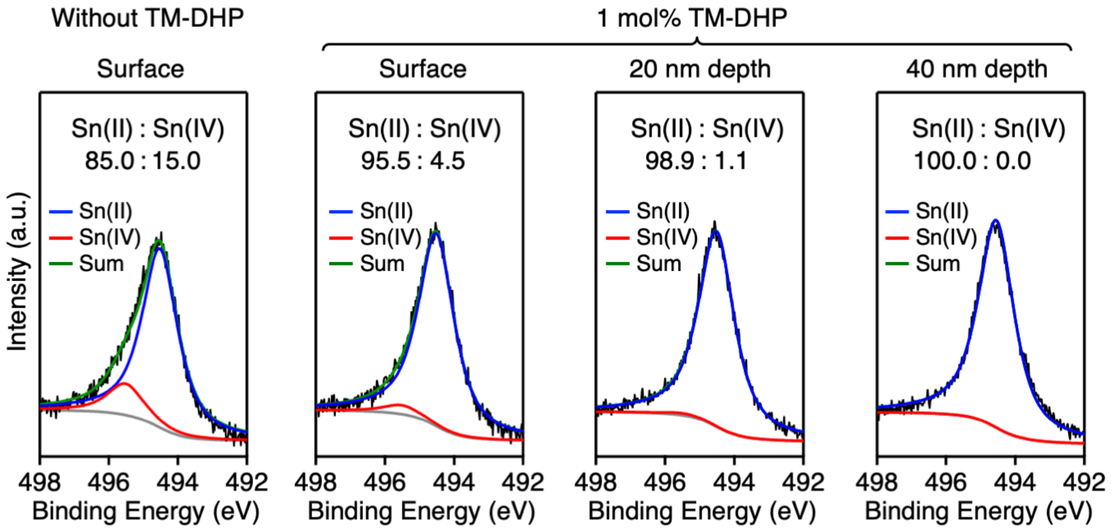

Supplementary Figure 8. XPS of Sn 3$d^{3/2}$ spectra of perovskite films. Experimental data, black line; Shirley background, gray line; Sn(II) peak fit, blue line; Sn(IV) peak fit, red line; sum of all deconvolution curves, green line.

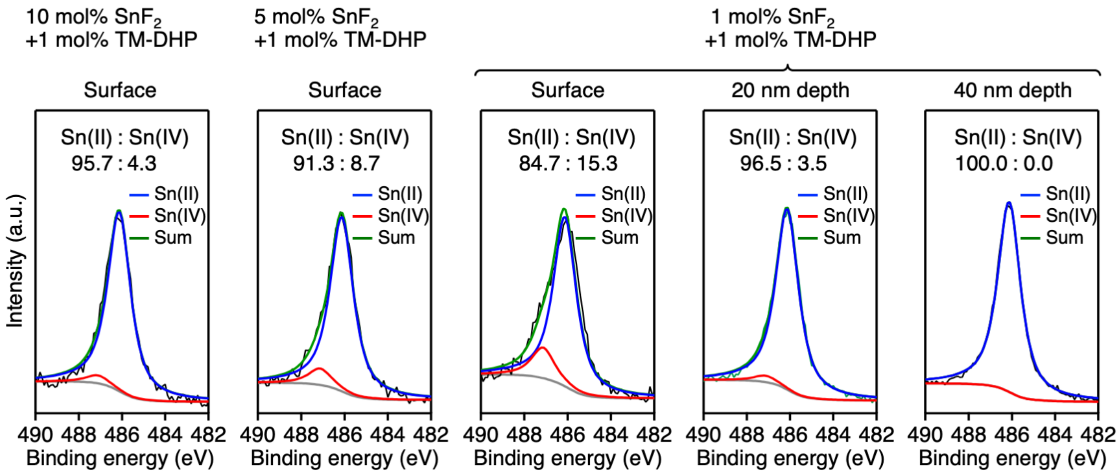

Supplementary Figure 9. XPS of Sn 3*d*$^{5/2}$ spectra of perovskite films. Experimental data, black line; Shirley background, gray line; Sn(II) peak fit, blue line; Sn(IV) peak fit, red line; sum of all deconvolution curves, green line.

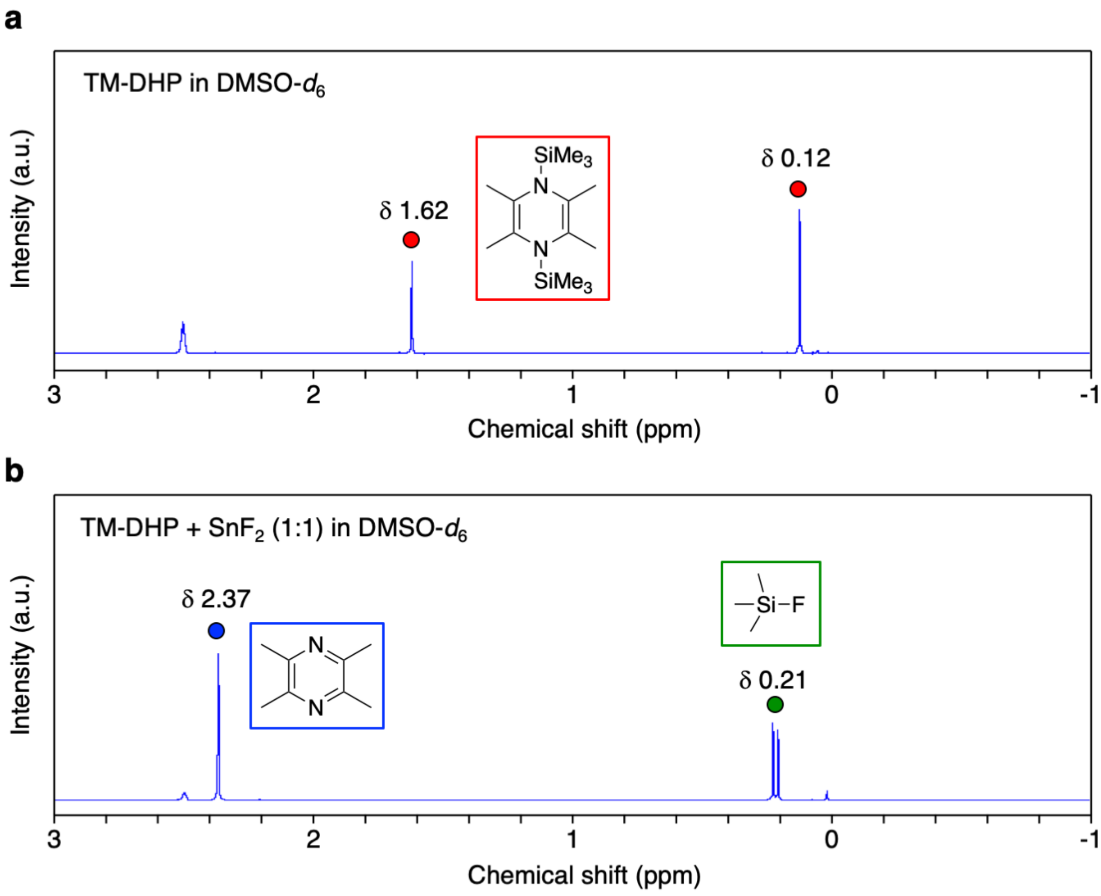

Supplementary Figure 10. Reaction of TM-DHP and SnF_2. ^{1}H NMR spectrum of **a** TM-DHP and **b** the mixture of TM-DHP and SnF_2 (1:1) in DMSO-d_6. A small peak at δ 0.09 is likely attributed to hexamethyldisilane which possibly formed by the reductive coupling of two trimethylsilyl species.

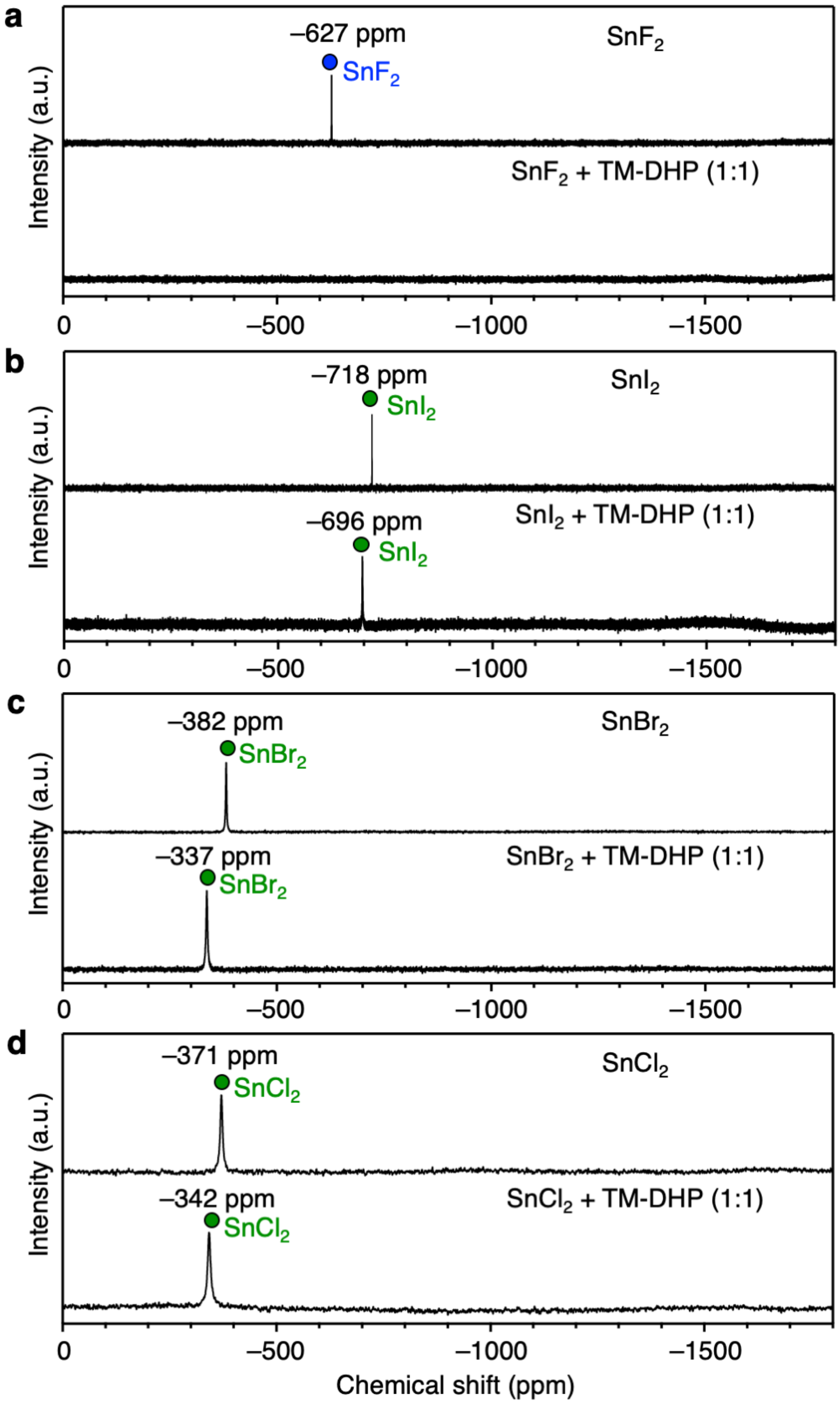

Supplementary Figure 11. Selective reaction of TM-DHP with SnF_2. ^{119}Sn NMR spectra of **a** SnF_2, **b** SnI_2, **c** $SnBr_2$, and **d** $SnCl_2$ in DMSO-d_6, before (top) and after (bottom) adding 1 equivalent of TM-DHP.

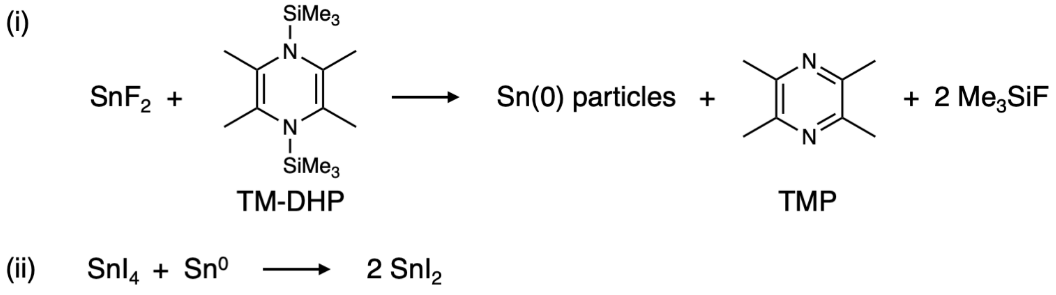

Supplementary Figure 12. Two-step reaction mechanism for the reduction of SnI_4 by TM-DHP and SnF_2.

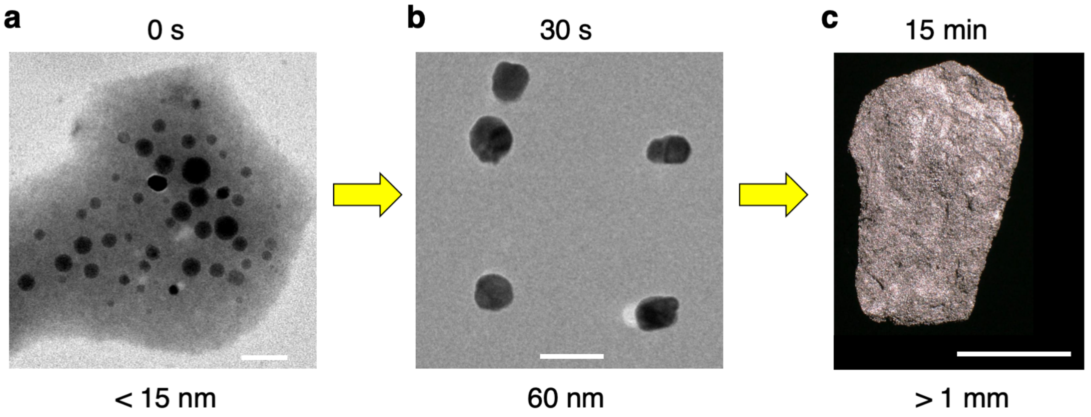

Supplementary Figure 13. Aggregation of Sn(0) nanoparticles. TEM images of the Sn(0) nanoparticles formed **a** just after the addition of TM-DHP (scale bar: 20 nm) and **b** after stirring for 30 s (scale bar: 100 nm). **c** Optical microscope image of the Sn(0) bulk metal formed after stirring for 15 min (scale bar: 1 mm).

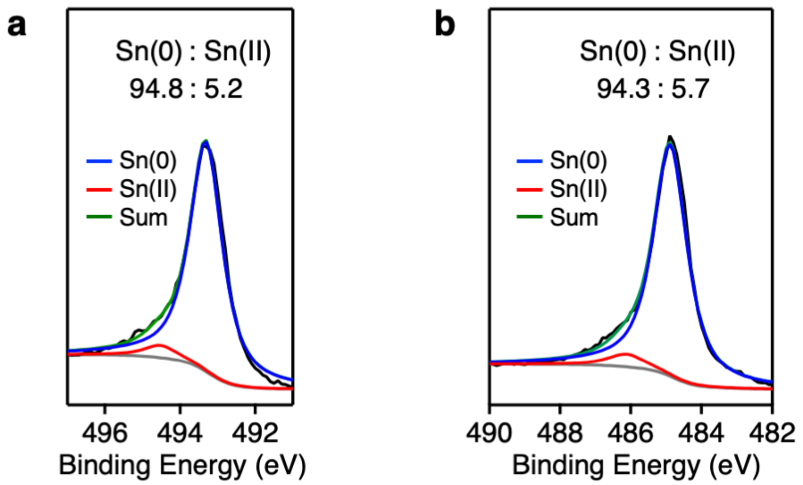

Supplementary Figure 14. Characterization of the metal precipitate. **a** Sn $3d^{3/2}$ and **b** Sn $3d^{5/2}$ XPS core levels of Sn(0) metal formed on the reaction of TM-DHP with SnF_2. Experimental data, black line; Shirley background, gray line; Sn(0) peak fit, blue line; Sn(II) peak fit, red line; sum of all deconvolution curves, green line.

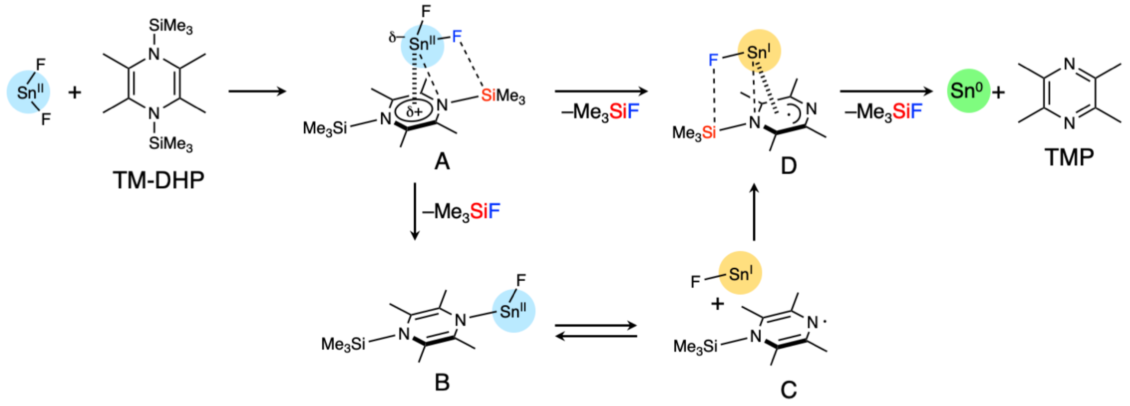

Supplementary Figure 15. Plausible reaction mechanism for the formation of Sn(0) nanoparticles. The electronic interaction between the tin center and TM-DHP forms intermediate **A**. The *N*-trimethylsilyl moiety reacts with the Sn–F bond and the subsequent elimination of Me_3SiF gives (4-trimethylsilyl-1,4-dihydropyrazine)tin species **B**, driven by the formation of stable Si–F bonds. High valent species **B** and organic radical **C** (stabilized by the delocalization of the unpaired electron in the six-membered ring) are in equilibrium. The organic radical **C** interacts with SnF to form intermediate **D**, which by the elimination of another Me_3SiF molecule forms Sn(0) metal and TMP.

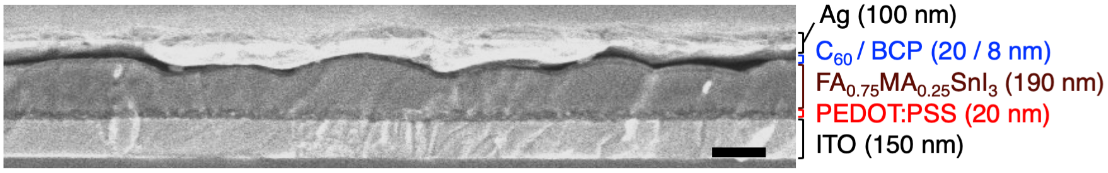

Supplementary Figure 16. Cross-sectional SEM image of a representative Sn-based PSC device with 1 mol% of TM-DHP. The scale bar is 200 nm.

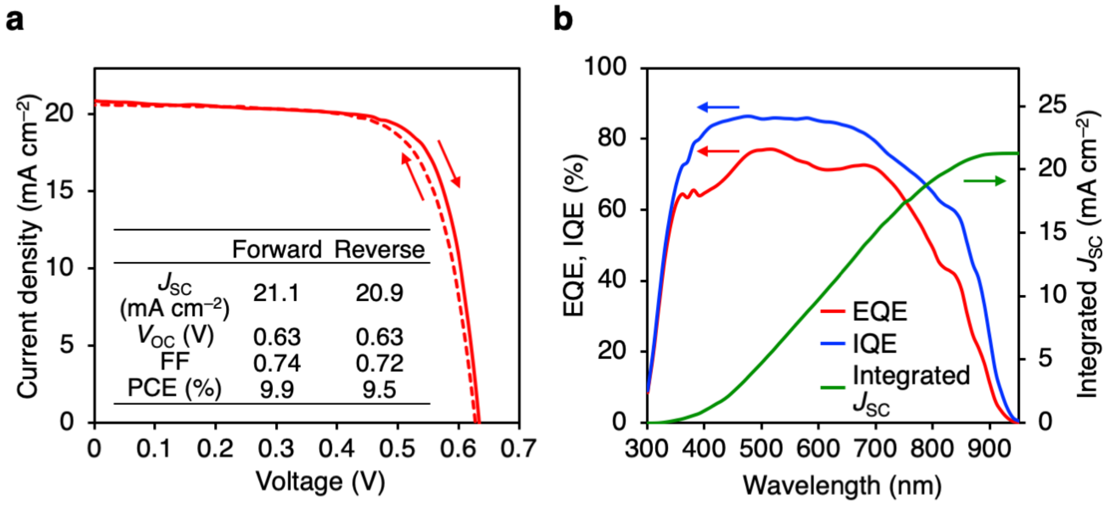

Supplementary Figure 17. Photovoltaic properties of the Sn perovskite-based solar cell devices. **a** Forward (solid line) and reverse (dashed line) *J*–*V* curves of the best device fabricated with 1 mol% of TM-DHP. **b** EQE and IQE spectra of a Sn-based PSC device fabricated with 1 mol% of TM-DHP.

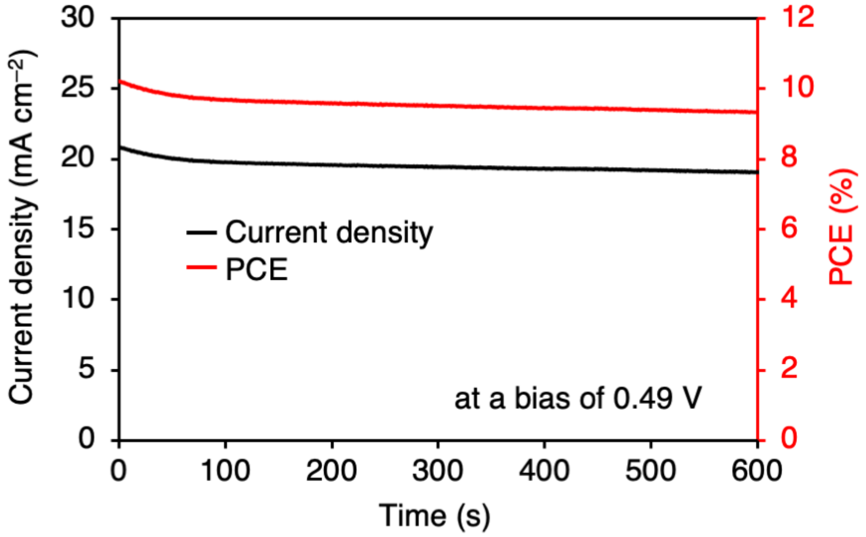

Supplementary Figure 18. Stabilized current density (black line) and stabilized power output (red line) of a representative $FA_{0.75}MA_{0.25}SnI_3$ perovskite solar cell measured at a bias of 0.49 V under AM 1.5G, 100 mW cm^{-2} irradiation.

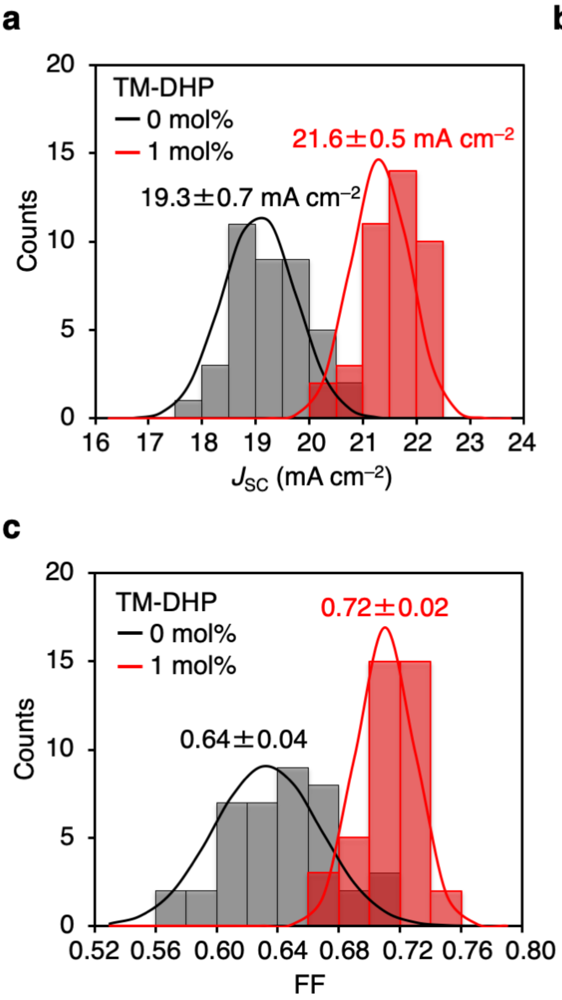

Supplementary Figure 19. Statistics of the **a** J_{SC}, **b** V_{OC}, and **c** FF distributions of 40 cells with or without 1 mol% of TM-DHP.

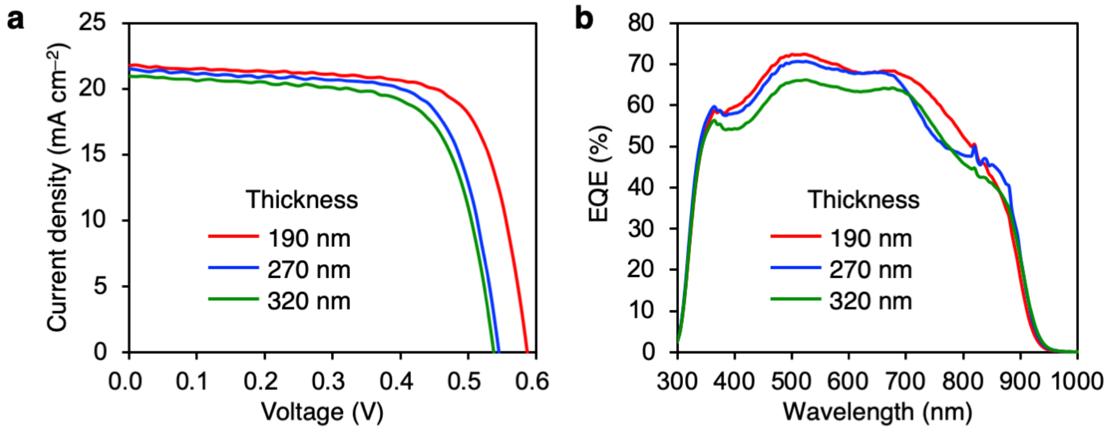

Supplementary Figure 20. Effect of perovskite thickness. **a** *J*–*V* curves and **b** EQE spectra of devices with perovskite layers of varying thicknesses.

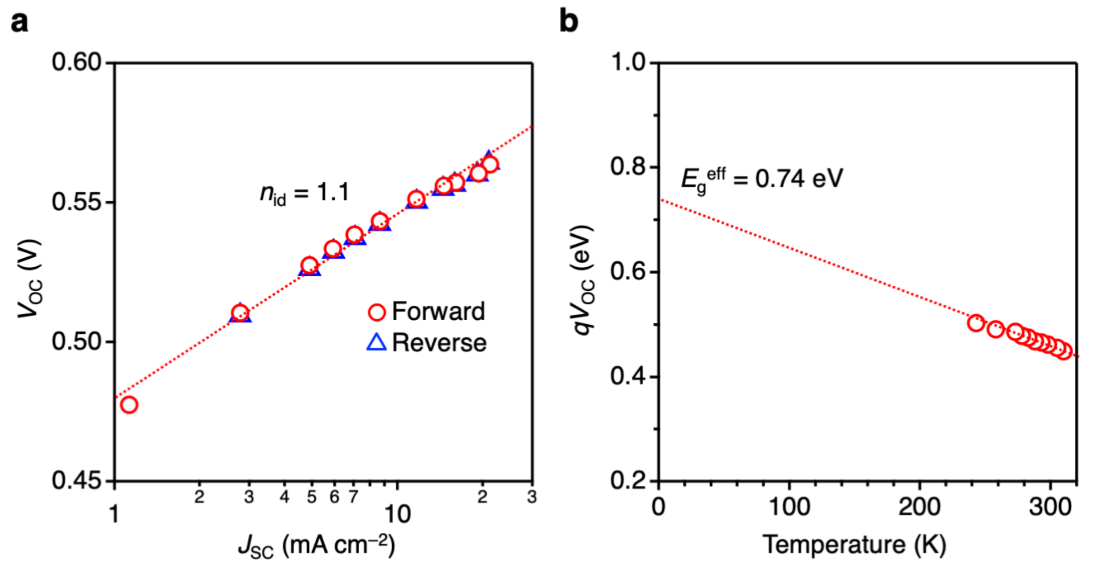

Supplementary Figure 21. Elucidation of the origin of voltage loss. **a** Light intensity and **b** temperature dependence of V_{OC} for Sn-based PSCs, from which the diode ideality factor (n_{id}) and effective band gap (E_g^{eff}) were estimated, respectively.

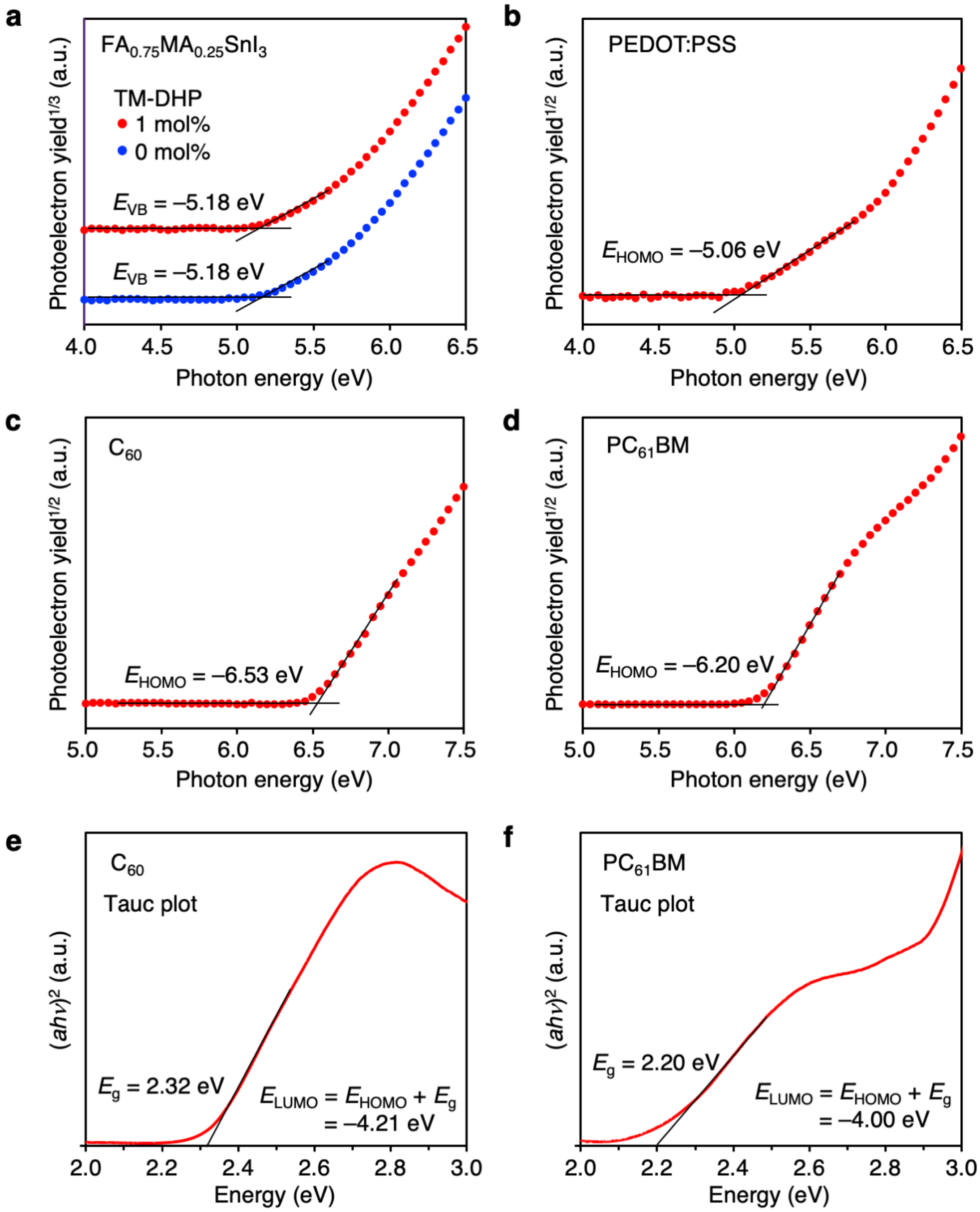

Supplementary Figure 22. Determination of energy levels. PYS spectra of **a** $FA_{0.75}MA_{0.25}SnI_3$ perovskite films prepared with or without 1 mol% of TM-DHP, **b** PEDOT:PSS, **c** C_{60}, and **d** $PC_{61}BM$ film. Tauc plot of the absorption spectrum of **e** C_{60} and **f** $PC_{61}BM$ film. Valence band energy (E_{VB}), HOMO energy (E_{HOMO}), and optical band gap (E_g) were estimated. Conduction band energy (E_{CB}) and LUMO energy (E_{LUMO}) were estimated by $E_{CB} = E_{VB} + E_g$ and $E_{LUMO} = E_{HOMO} + E_g$, respectively.

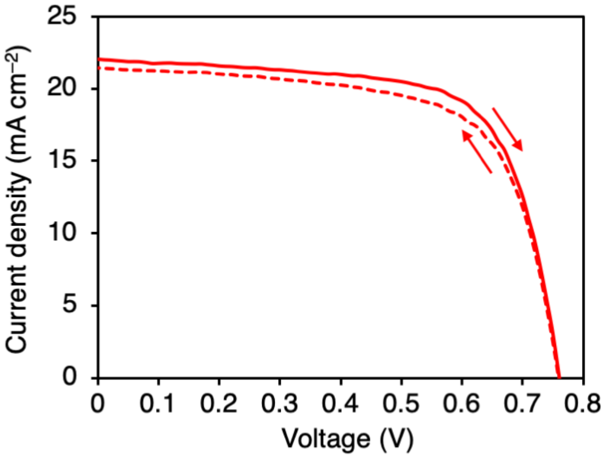

	Forward	Reverse
J_{SC} (mA cm^{-2})	22.0	21.5
V_{OC} (V)	0.76	0.76
FF	0.69	0.67
PCE (%)	11.5	10.9

Supplementary Figure 23. Forward (solid line) and reverse (dashed line) *J*–*V* curves of the best device treated with EDA and $PC_{61}BM$.

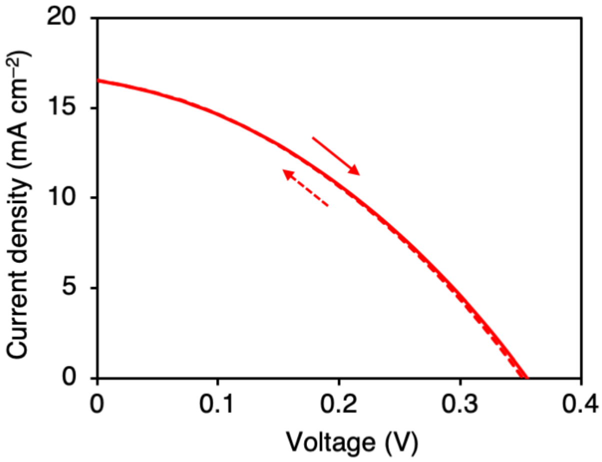

	Forward	Reverse
J_{SC} (mA cm^{-2})	16.5	16.5
V_{OC} (V)	0.36	0.35
FF	0.36	0.37
PCE (%)	2.1	2.1

Supplementary Figure 24. Forward (solid line) and reverse (dashed line) *J*–*V* curves of the device fabricated with no C_{60} layer.

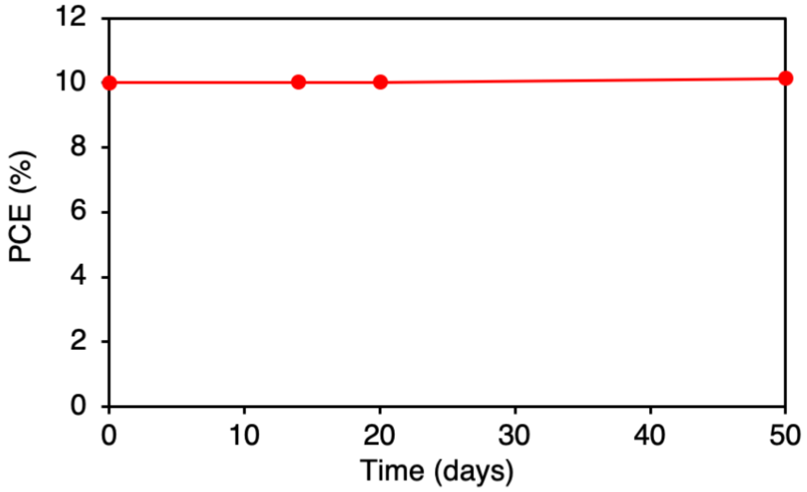

Supplementary Figure 25. Stability of the device treated with EDA and $PC_{61}BM$ when stored and measured in N_2 atmosphere at ambient temperature.

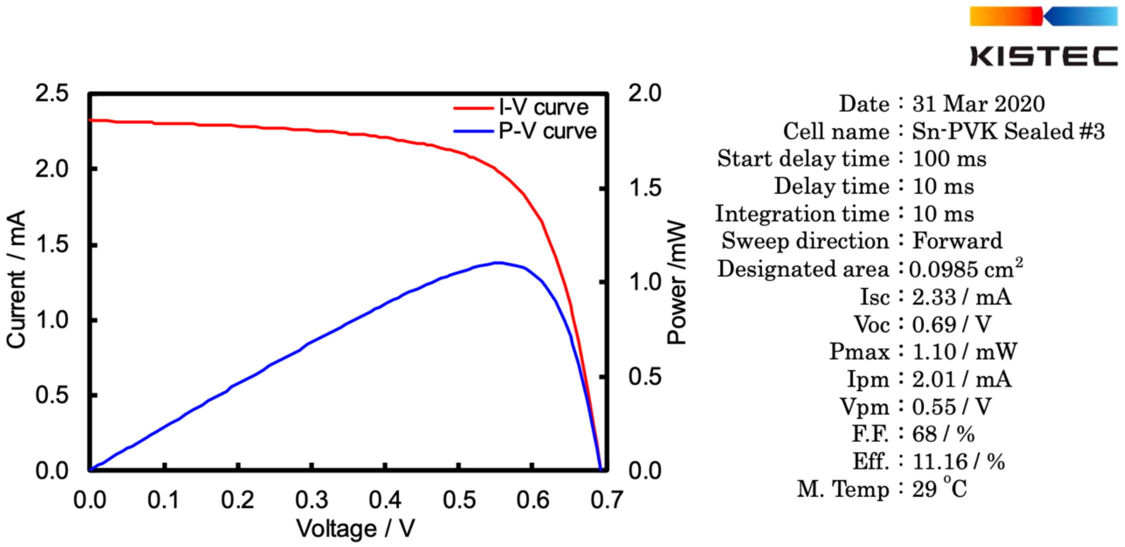

Supplementary Figure 26. Certification data of an encapsulated device, measured by the Kanagawa Institute of Industrial Science and Technology (KISTEC).

Supplementary Note 1:

Synthesis of 2,3,5,6-tetramethyl-1,4-bis(dimethylsilyl)-1,4-dihydropyrazine (TM-DHP)

In a two-necked 100 mL flask, 2,3,5,6-tetramethylpyrazine (1.36 g, 10 mmol, Tokyo Chemical Industry Co.) was dissolved in THF (50 mL, Kanto Chemical. Co.) and clean-cut potassium (1.44 g, 37 mmol, Kanto Chemical. Co.) and Me_3SiCl (3.8 mL, 30 mmol, Tokyo Chemical Industry Co.) were added, in this order. After stirring 24 h at room temperature, the reaction mixture was filtered and the most of the solvent was removed by evaporation. Obtained oil was purified by sublimation under reduced pressure (30 Pa) up to 100 °C using Kugelrohr distillation apparatus. The target compound (2.12 g, 7.5 mmol, 75%) was obtained as colorless crystals. ^1H NMR (400 MHz, C_6D_6): δ 1.69 (s, 12H), 0.23 (s, 18H), which corresponds to the literature.[1]

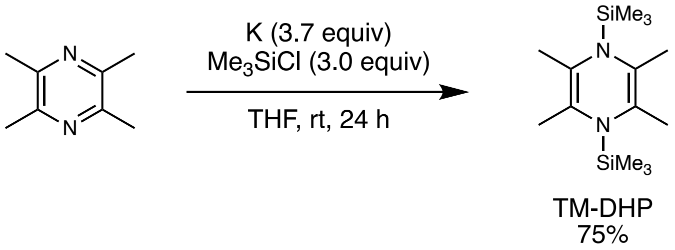

Supplementary Figure 27. Synthesis of TM-DHP.

Supplementary Note 2:

X-ray Crystal Structure Analysis

Single crystals of 2,3,5,6-tetramethyl-1,4-bis(dimethylsilyl)-1,4-dihydropyrazine (TM-DHP) suitable for X-ray diffraction analysis were obtained by sublimation using Kugelrohr distillation apparatus. Diffraction data was collected at 100 K on a Bruker Single-Crystal CCD X-ray Diffractometer (SMART APEX II ULTRA) with Mo Kα radiation (λ = 0.71073 Å) and graphite monochromater. A total of 3351 reflections were measured with a maximum 2θ angle of 51.0°, of which 1266 were independent reflections (R_{int} = 0.995). The structure was solved by direct methods (SHELXS-97[2]) and refined by the full-matrix least-squares on F^2 (SHELXL-97). All non-hydrogen atoms were refined anisotropically. All hydrogen atoms were placed using AFIX instructions. The crystal data are as follows: $C_{14}H_{30}N_2Si_2$; FW = 282.57, crystal size 0.20 × 0.21 × 0.22 mm, Triclinic, *P*-1, a = 9.361(2) Å, b = 14.316(4) Å, c = 14.526(4) Å, α = 89.598(4)°, β = 72.102(3)°, γ = 74.288(3)°, V = 1777.1(8)Å^3, Z = 2, D_c = 1.054 g cm^{-3}. The refinement converged to R_1 = 0.0456, wR_2 = 0.1412 ($I > 2\sigma(I)$), GOF = 0.974. Crystallographic data have been deposited with the Cambridge Crystallographic Data Center as supplementary publication no. CCDC-1979950 (TM-DHP). These data can be obtained free of charge from The Cambridge Crystallographic Data Centre via www.ccdc.cam.ac.uk/data_request/cif.

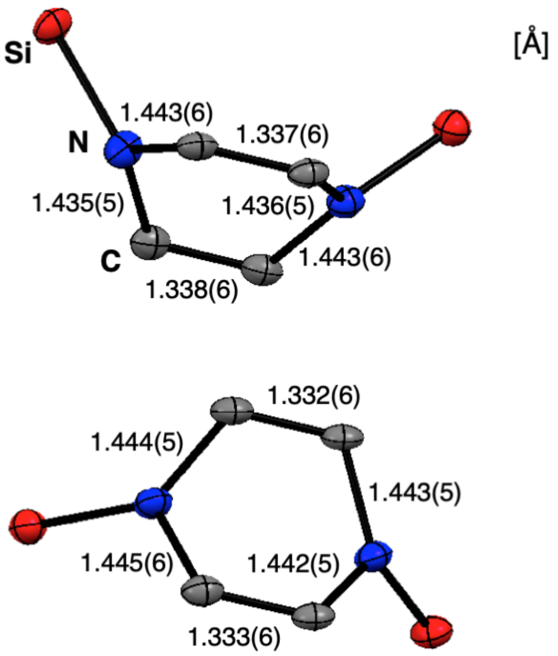

Supplementary Figure 28. ORTEP drawing (thermal ellipsoids at 50% probability) of TM-DHP. The methyl groups are omitted for clarity.

Supplementary References

1. Kaim, W. Effects of cyclic 8-π-electron conjugation in reductively silylated N-heterocycles. *J. Am. Chem. Soc.* **105**, 707–713 (1983).
2. Sheldrick, G. M. SHELX-97, Program for the Refinement of Crystal Structures; University of Göttingen: Göttingen, Germany, 1997.
