# Supplementary material for: Sn(IV)-free tin perovskite films realized by in situ Sn(0) nanoparticle treatment of the precursor solution
Source: Nat Commun. 2020 Jun 16;11:3008. doi: 10.1038/s41467-020-16726-3 (PMC7297727; doi:10.1038/s41467-020-16726-3)
Supplement: Supplementary file 3 — Solar Cells Reporting Summary [file 41467_2020_16726_MOESM3_ESM.pdf]

nature research

Corresponding author(s): Atsushi Wakamiya

Solar Cells Reporting Summary

Nature Research wishes to improve the reproducibility of the work that we publish. This form is intended for publication with all accepted papers reporting the characterization of photovoltaic devices and provides structure for consistency and transparency in reporting. Some list items might not apply to an individual manuscript, but all fields must be completed for clarity.

For further information on Nature Research policies, including our data availability policy, see Authors & Referees.

ü Experimental design

Please check: are the following details reported in the manuscript?

1. Dimensions

Item	Yes	No	Location / explanation
Area of the tested solar cells	☒	☐	Methods; Characterizations
Method used to determine the device area	☒	☐	Methods; Characterizations

2. Current-voltage characterization

Item	Yes	No	Location / explanation
Current density-voltage (J-V) plots in both forward and backward direction	☒	☐	Supplementary Information Figures 17, 23, & 24
Voltage scan conditions<br>*For instance: scan direction, speed, dwell times*	☒	☐	Methods; Characterizations
Test environment<br>*For instance: characterization temperature, in air or in glove box*	☒	☐	Methods; Characterizations
Protocol for preconditioning of the device before its characterization	☐	☒	No preconditioning method was used.
Stability of the J-V characteristic<br>*Verified with time evolution of the maximum power point or with the photocurrent at maximum power point; see ref. 7 for details.*	☒	☐	Supplementary Information Figure 18

3. Hysteresis or any other unusual behaviour

Item	Yes	No	Location / explanation
Description of the unusual behaviour observed during the characterization	☒	☐	Minor hysteresis (Supplementary Information Figures 17, 23, & 24)
Related experimental data	☐	☒	No significant difference in PCE for forward and reverse scans

4. Efficiency

Item	Yes	No	Location / explanation
External quantum efficiency (EQE) or incident photons to current efficiency (IPCE)	☒	☐	Figure 5 & Supplementary Information Figure 17
A comparison between the integrated response under the standard reference spectrum and the response measure under the simulator	☒	☐	Figure 5 & Supplementary Information Figure 17
For tandem solar cells, the bias illumination and bias voltage used for each subcell	☐	☒	No tandem solar cells were evaluated.

5. Calibration

Item	Yes	No	Location / explanation
Light source and reference cell or sensor used for the characterization	☒	☐	Methods; Characterizations
Confirmation that the reference cell was calibrated and certified	☒	☐	Methods; Characterizations

Calculation of spectral mismatch between the reference cell and the devices under test

- [ ] Yes
- [x] No

The solar simulators are 3A grade

6. Mask/aperture

Size of the mask/aperture used during testing

- [x] Yes
- [ ] No

Methods; Characterizations

Variation of the measured short-circuit current density with the mask/aperture area

- [ ] Yes
- [x] No

They were already measured at the maximum aperture

7. Performance certification

Identity of the independent certification laboratory that confirmed the photovoltaic performance

- [x] Yes
- [ ] No

Kanagawa Institute of Industrial Science and Technology (KISTEC), Japan

A copy of any certificate(s)
Provide in Supplementary Information

- [x] Yes
- [ ] No

Supplementary Information Figure 26

8. Statistics

Number of solar cells tested

- [x] Yes
- [ ] No

Table 1 & Table 2

Statistical analysis of the device performance

- [x] Yes
- [ ] No

Table 1 & Table 2

9. Long-term stability analysis

Type of analysis, bias conditions and environmental conditions
For instance: illumination type, temperature, atmosphere humidity, encapsulation method, preconditioning temperature

- [x] Yes
- [ ] No

Supplementary Information Figure 25
